# Association of ultrasound-derived fat fraction with carotid intima-media thickness and carotid plaque in patients with type 2 diabetes: an exploratory cross-sectional study

**DOI:** 10.3389/fendo.2026.1940570

**Published:** 2026-09-25

**Authors:** Zheng Hua, Shuangshuang Zhao, Zheng Zhang, Yanwei Chen, Minfang Yao, Mengjia Xia, Mengfan Yang, Xin Zhang

**Affiliations:** 1 Department of Ultrasound Medicine, Affiliated Hospital of Jiangsu University, Zhenjiang, Jiangsu, China; 2 Department of Medical Ultrasound, Kunshan Second People’s Hospital, Suzhou, China

**Keywords:** carotid intima-media thickness, carotid plaque, hepatic steatosis, type 2 diabetes mellitus, ultrasound-derived fat fraction

## Abstract

**Objective:**

To investigate the associations of continuous ultrasound-derived fat fraction (UDFF) with carotid intima-media thickness (CIMT) and prevalent carotid plaque in patients with type 2 diabetes mellitus (T2DM), assess the incremental statistical information provided by UDFF in primary and broader traditional vascular-risk adjustment models, and compare its within-cohort discrimination with conventional qualitative liver ultrasonography.

**Methods:**

This single-center retrospective cross-sectional study included 153 patients with T2DM evaluated in the Department of Endocrinology, Affiliated Hospital of Jiangsu University, between September and December 2025. Continuous UDFF was the primary exposure. CIMT was analyzed using linear regression and prevalent carotid plaque using logistic regression. The primary multivariable model adjusted for age, sex, statin therapy, and low-density lipoprotein cholesterol (LDL-C).

**Results:**

Each 1-percentage-point increase in UDFF was associated with a 0.0105-mm higher CIMT (95% CI, 0.0080–0.0131; P<0.001) and 10.9% higher odds of prevalent carotid plaque (OR = 1.109, 95% CI, 1.056–1.164; P<0.001) after multivariable adjustment. Associations remained materially similar in broader covariate models and after excluding 28 participants with prior atherosclerotic cardiovascular disease. Adding UDFF to the primary clinical adjustment model increased R² for CIMT from 0.274 to 0.498 and the C-statistic for carotid plaque from 0.633 to 0.747 (DeLong P = 0.018). In the broader traditional vascular-risk model, the C-statistic increased from 0.697 to 0.758 (ΔC-statistic=0.061; DeLong P = 0.045). In otherwise identical clinical models, discrimination for prevalent carotid plaque was greater with continuous UDFF than with the original four-category conventional ultrasonographic steatosis grade (0.747 vs 0.657; DeLong P = 0.008).

**Conclusion:**

Higher UDFF was associated with greater CIMT and prevalent carotid plaque in patients with T2DM. UDFF provided incremental statistical information within this cohort, although the magnitude of improvement in plaque discrimination was smaller in the broader traditional vascular-risk model than in the primary clinical adjustment model. Continuous UDFF also showed greater within-cohort discrimination than conventional four-category ultrasonographic steatosis grading. These cross-sectional findings do not establish causality or prospective cardiovascular risk prediction.

## 1 Introduction

Hepatic steatosis is common in patients with type 2 diabetes mellitus (T2DM) and often coexists with obesity, insulin resistance, dyslipidemia, and hypertension (1–4). Because these conditions are also major determinants of atherosclerosis, it remains uncertain whether hepatic fat itself contributes to vascular injury or mainly reflects the underlying metabolic burden (1, 2, 5). This distinction is important when assessing whether a quantitative measure of hepatic fat provides information beyond established cardiovascular risk factors.

Carotid ultrasonography provides a noninvasive means of characterizing vascular structural changes. Carotid intima-media thickness (CIMT) and carotid plaque represent related but distinct carotid phenotypes (6, 7). CIMT is a continuous measure of arterial wall thickness that is influenced by age and cardiovascular risk factors (8), whereas carotid plaque represents focal atherosclerotic change and has been associated with cardiovascular risk (6, 7). Importantly, current recommendations emphasize carotid plaque for ultrasound-based atherosclerotic risk characterization, while the interpretation of CIMT depends on acquisition technique and the population studied, and the use of a single universal threshold may be problematic. Accordingly, continuous CIMT was evaluated together with prevalent carotid plaque (9).

Conventional B-mode ultrasonography is widely used to assess hepatic steatosis, but visual grading is qualitative and subject to interobserver variation (10–12). UDFF combines attenuation and backscatter information to provide a quantitative estimate of hepatic fat fraction (13, 14). Previous studies have reported good agreement between UDFF and MRI-PDFF and favorable interobserver reproducibility (15, 16). UDFF does not diagnose steatohepatitis or hepatic fibrosis, and in the absence of MRI-PDFF or histologic validation, it was treated in the present study as a quantitative ultrasound estimate rather than a reference-standard measure of hepatic fat content (13, 15–18).

Most studies linking hepatic steatosis with carotid disease have relied on qualitative liver ultrasonography or surrogate steatosis indices (19–21). A recent prospective study reported an association between UDFF and panvascular disease in patients with MASLD, but it did not specifically examine carotid structural changes in patients with T2DM (22). It remains unclear whether continuous UDFF is associated with both CIMT and carotid plaque in this population and whether it provides information beyond conventional qualitative liver ultrasonography. We therefore examined the associations of continuous UDFF with CIMT and prevalent carotid plaque, explored its incremental statistical information in primary and broader traditional vascular-risk adjustment models, and compared its within-cohort discrimination with conventional qualitative liver ultrasonography.

## 2 Materials and methods

### 2.1 Study design and participants

This retrospective cross-sectional study included patients with type 2 diabetes mellitus (T2DM) who were evaluated in the Department of Endocrinology, Affiliated Hospital of Jiangsu University, between September and December 2025. Participants were retrospectively identified from the electronic medical record, laboratory information system, and stored ultrasound databases. Laboratory measurements and the relevant ultrasound examinations were required to have been obtained either on the day before admission or on the first day of hospitalization. For all participants, the interval among UDFF assessment, carotid ultrasonography, and laboratory testing did not exceed 24 hours. No formal *a priori* sample-size calculation was performed; the sample size was determined by the number of eligible patients available during the study period.

T2DM was identified based on a documented clinical diagnosis recorded in the electronic medical records, including previous medical history and admission diagnosis. UDFF assessment, carotid ultrasonography, and laboratory testing had been performed as part of routine clinical care rather than specifically for the purposes of this study.

Patients were excluded if they met any of the following criteria: age <18 years; acute diabetic complications; serological evidence of HBV or HCV infection, defined as HBsAg positivity or reactive anti-HCV screening; documented excessive alcohol consumption; severe renal insufficiency; thyroid or parathyroid disorders; autoimmune disease; malignancy; pregnancy; psychiatric conditions precluding completion of the relevant clinical examinations. Availability of corresponding inpatient clinical and laboratory records was required for inclusion in the analytic cohort. Availability of the key clinical, laboratory, and ultrasound variables required for the primary analyses was required for inclusion in the final analytic cohort. Patients with substantial missing key clinical or laboratory data were excluded before construction of the final analytic dataset.

The study was approved by the Ethics Committee of Affiliated Hospital of Jiangsu University (Approval No. KY2025K0505). The requirement for informed consent was waived by the Ethics Committee. This study was reported in accordance with the Strengthening the Reporting of Observational Studies in Epidemiology (STROBE) statement for cross-sectional studies (23).

### 2.2 Clinical and laboratory measurements

Demographic characteristics, anthropometric measurements, diabetes duration, smoking and alcohol-use status, medication use, medical history, and laboratory results were retrospectively extracted from the medical records. Clinical ASCVD was defined as a documented history of coronary artery disease, myocardial infarction, ischemic stroke/transient ischemic attack, peripheral arterial disease, or prior coronary/carotid revascularization. Height and weight were measured during routine clinical assessment, and BMI was calculated as weight in kilograms divided by height in meters squared. Waist and hip circumferences were recorded, and the waist-to-hip ratio was calculated as waist circumference divided by hip circumference.

Systolic blood pressure (SBP) and diastolic blood pressure (DBP) were obtained from the blood-pressure measurements recorded at admission. Current antihypertensive, statin, and glucose-lowering therapies were extracted from the medical records. Detailed information on individual glucose-lowering drug classes, dose, duration, and adherence was incomplete and was therefore not analyzed.

Only laboratory measurements obtained within the defined measurement window were used. Laboratory variables included fasting plasma glucose (FPG), 2-hour postprandial glucose (2-h PG), fasting insulin (FIns), fasting C-peptide, glycated hemoglobin (HbA1c), triglycerides (TG), total cholesterol (TC), high-density lipoprotein cholesterol (HDL-C), low-density lipoprotein cholesterol (LDL-C), alanine aminotransferase (ALT), aspartate aminotransferase (AST), γ-glutamyl transferase (GGT), and uric acid (UA). The homeostasis model assessment of insulin resistance (HOMA-IR) was calculated as fasting plasma glucose (mmol/L) × fasting insulin (μU/mL)/22.5 (24). HOMA-IR was used descriptively and was not included in the primary multivariable models because fasting insulin may be influenced by ongoing glucose-lowering therapy.

### 2.3 Liver ultrasonography, UDFF, and shear-wave measurements

Liver ultrasonography was performed using a Siemens ACUSON Sequoia ultrasound system equipped with a deep abdominal convex (DAX) transducer. The liver and carotid ultrasound examinations were performed by different radiologists as part of routine clinical care. During the same routine clinical liver ultrasound examination, conventional B-mode assessment was performed first, followed by UDFF acquisition using the same ultrasound system. Patients were examined in the supine position, and the right hepatic lobe was visualized through an intercostal approach. The region of interest was positioned within the right hepatic lobe while avoiding large vessels, bile ducts, focal lesions, and rib shadowing. Five valid UDFF measurements were available for each participant, and the median of the five measurements was used as the participant-level UDFF value. For the present analysis, UDFF values were categorized as S0 (<8%), S1 (8% to <14%), S2 (14% to <20%), or S3 (≥20%) (25). Continuous UDFF was retained as the primary exposure variable, whereas UDFF grades were used for descriptive and ordered-trend analyses. Because the source study also proposed dual rule-out/rule-in thresholds with intervening indeterminate zones, these single-cutoff categories were used descriptively and were not interpreted as definitive diagnostic steatosis grades (25). During the five UDFF acquisitions, Auto point shear-wave elastography (Auto pSWE) was obtained simultaneously, and an acquisition-specific median shear-wave velocity (Vs Median, m/s) was generated by the ultrasound system. For each participant, the median of the five acquisition-specific Vs Median values was used as the participant-level liver shear-wave velocity.

### 2.4 Conventional qualitative liver ultrasonography

Conventional hepatic steatosis was qualitatively graded as absent, mild, moderate, or severe by experienced radiologists during routine B-mode ultrasonography. Grading was based on standard sonographic features, including increased hepatic echogenicity relative to the renal cortex, posterior beam attenuation, and reduced visualization of intrahepatic vessels and the diaphragm (10, 26). For comparison with UDFF, conventional ultrasonographic steatosis was primarily modeled using its original four-category grading (absent, mild, moderate, and severe), with absent steatosis as the reference category. The original qualitative grade recorded in the routine clinical ultrasound report was used for analysis and was not reassigned for the present study. Alternative ordinal (0–3) and binary (absent vs any steatosis) codings were evaluated in sensitivity analyses.

### 2.5 Carotid ultrasonography

Carotid ultrasonography was performed as part of routine clinical care using the same ultrasound platform equipped with a linear-array transducer. Bilateral common carotid arteries (CCA), carotid bifurcations, and internal carotid arteries (ICA) were examined in longitudinal and transverse planes by experienced radiologists.

CIMT was measured at the far wall of the common carotid artery approximately 1 cm proximal to the carotid bulb. Three measurements were obtained on each side, and the mean of the six measurements was used as the participant-level CIMT value. Continuous CIMT served as the primary carotid wall-thickness outcome. For descriptive and exploratory secondary analyses, CIMT thickening was defined as a mean CIMT ≥1.0 mm (27). Carotid plaque was defined as a focal structure encroaching into the arterial lumen by at least 0.5 mm or 50% of the surrounding intima-media thickness, or demonstrating a thickness >1.5 mm measured from the media-adventitia interface to the intima-lumen interface (6). The presence of at least one plaque in the CCA, carotid bifurcation, or ICA on either side was classified as prevalent carotid plaque. Representative liver and carotid ultrasound images are shown in **Figure 1**.

**Figure 1:**
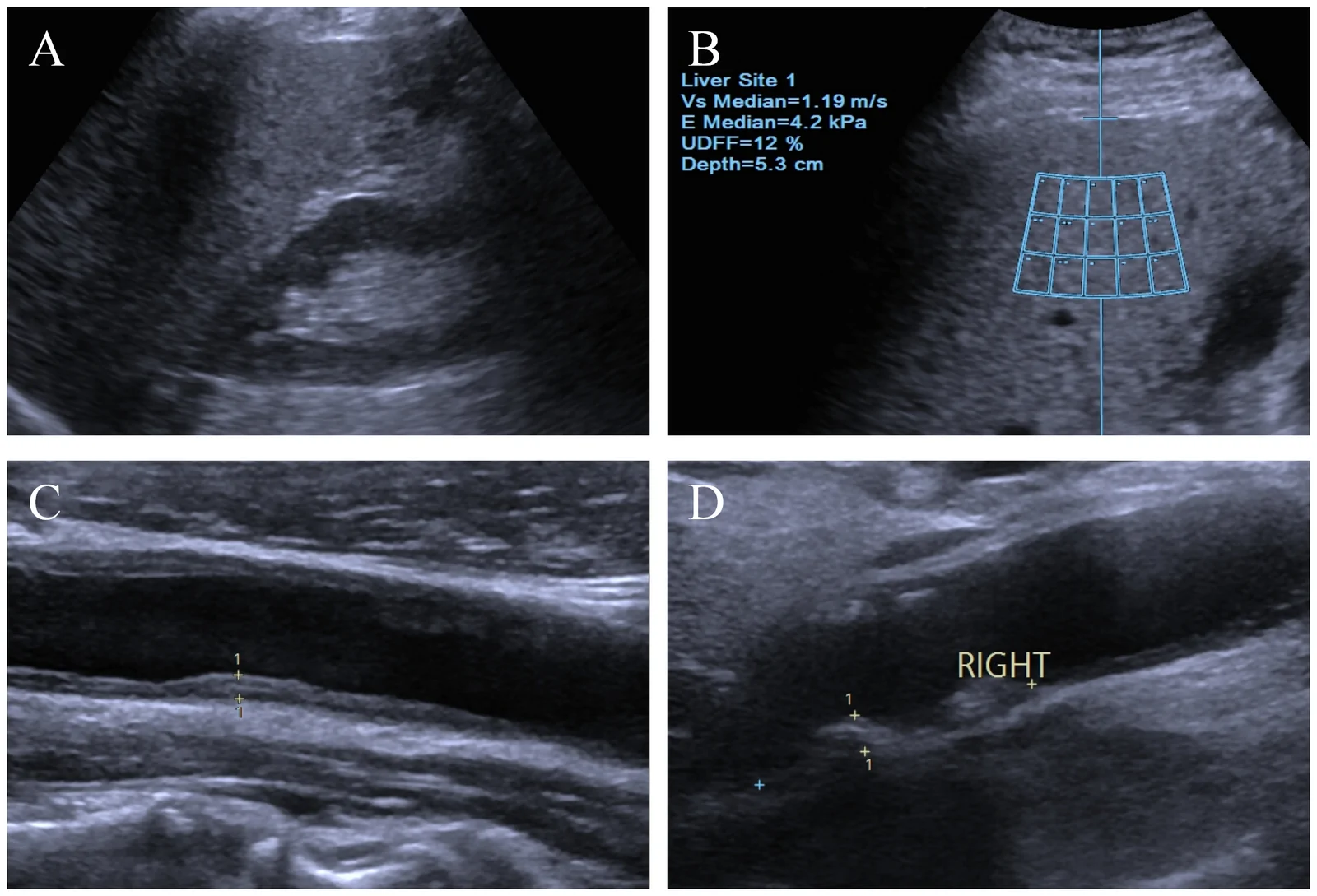
Representative ultrasound images of liver and carotid assessments. **(A)** Conventional B-mode ultrasonographic image of the liver. **(B)** Representative simultaneous UDFF and Auto pSWE acquisition showing placement of the measurement region within the right hepatic lobe. The displayed depth indicates the distance from the skin surface to the measurement region. The display includes UDFF together with Vs Median (m/s) and E Median (kPa); liver shear-wave velocity (Vs) was used as the supplementary liver-stiffness measure in the present analysis. **(C)** Representative carotid ultrasonographic image showing measurement of CIMT. **(D)** Representative carotid ultrasonographic image demonstrating carotid plaque. UDFF, ultrasound-derived fat fraction; CIMT, carotid intima-media thickness.

### 2.6 Inter-reader reanalysis agreement

For the assessment of inter-reader reanalysis agreement, 30 examinations were selected from the final stored imaging dataset after completion of the primary dataset. Each participant was assigned a unique study identification number, and a computer-generated random sequence was used for selection independently of UDFF and CIMT values. No participant was recalled or underwent a repeat ultrasound examination; both readers evaluated data from the same original stored examinations. For UDFF, the second reader independently repositioned the region of interest on each of the five stored acquisitions, repeated the measurements, and calculated the median of the five values as the participant-level UDFF value. For CIMT, the second reader independently repeated the measurements on the same stored carotid ultrasound images and recalculated the participant-level mean CIMT.

Inter-reader agreement for the resulting participant-level UDFF and CIMT values was evaluated using a two-way random-effects, absolute-agreement, single-measurement intraclass correlation coefficient [ICC (2,1)/ICC(A,1)] (28) and Bland–Altman analysis. Agreement for carotid plaque classification was not reassessed because the archived examinations did not consistently contain complete standardized sweeps of all carotid segments required to independently establish plaque absence.

### 2.7 Statistical analysis

Continuous variables were summarized as median (interquartile range), and categorical variables as number (percentage). Comparisons across UDFF grades were performed using the Kruskal-Wallis test for continuous variables. Categorical variables were compared using Pearson χ² tests when expected cell counts were adequate; Fisher-Freeman-Halton exact tests were used for multi-category variables with sparse expected counts. For ordered UDFF categories, trends in CIMT and carotid plaque prevalence were assessed using the Jonckheere-Terpstra and Cochran-Armitage tests, respectively. The Kruskal-Wallis comparison of CIMT was followed by Dunn pairwise comparisons with Holm adjustment. As a sensitivity analysis for multiple testing, P values from the exploratory between-grade comparisons in **Table 1** were additionally adjusted using the Benjamini-Hochberg false-discovery-rate (FDR) procedure. As a sensitivity analysis of the categorical findings, participants within the dual-threshold indeterminate zones were excluded separately for each steatosis boundary, and CIMT and plaque prevalence were compared between the corresponding rule-out and rule-in groups.

**Table 1:** Clinical, biochemical, and ultrasound characteristics according to UDFF grade.

Variable	Total (N = 153)	S0 (n=51)	S1 (n=36)	S2 (n=27)	S3 (n=39)	P value
UDFF, %	11.0 (6.0-20.0)	5.0 (4.0-6.0)	10.0 (9.0-12.0)	17.0 (14.5-17.5)	28.0 (24.5-32.0)	<0.001
Liver shear-wave velocity, m/s	1.22 (1.04-1.35)	1.26 (1.12-1.38)	1.13 (0.96-1.30)	1.20 (1.06-1.39)	1.15 (1.04-1.29)	0.274
CIMT, mm	0.85 (0.74-1.05)	0.80 (0.70-0.90)	0.85 (0.75-1.01)	0.85 (0.65-1.00)	1.10 (0.95-1.20)	<0.001
CIMT thickening, n (%)	54 (35.3)	7 (13.7)	10 (27.8)	9 (33.3)	28 (71.8)	<0.001
Carotid plaque, n (%)	89 (58.2)	18 (35.3)	20 (55.6)	19 (70.4)	32 (82.1)	<0.001
Age, years	60 (51–69)	60 (50–68)	62 (53–69)	58 (42-66)	60 (54-73)	0.353
BMI, kg/m²	25.00 (23.10-27.39)	23.34 (21.73-25.10)	24.95 (23.34-26.90)	26.56 (24.68-29.79)	26.73 (23.80-30.66)	<0.001
Waist circumference, cm	93 (88-100)	90 (85-96)	91 (86-97)	96 (92-106)	100 (90-106)	<0.001
Hip circumference, cm	97 (92-103)	96 (90-100)	96 (92-101)	98 (95-104)	102 (96-108)	<0.001
Waist-to-hip ratio	0.958 (0.916-0.989)	0.938 (0.898-0.985)	0.957 (0.915-0.979)	0.962 (0.929-1.000)	0.964 (0.933-1.001)	0.081
SBP, mmHg	150 (133-167)	148 (131-168)	150 (132-162)	157 (132-160)	150 (145-168)	0.818
DBP, mmHg	88 (78-96)	80 (76-90)	87 (77-93)	90 (80-100)	92 (82-100)	0.003
Diabetes duration, years	6.0 (1.0-13.0)	5.0 (0.4-10.0)	3.0 (0.6-10.0)	4.0 (0.7-9.0)	12.0 (7.0-20.0)	<0.001
FPG, mmol/L	9.72 (7.32-12.58)	8.86 (6.44-11.72)	9.25 (7.77-12.57)	10.27 (7.23-13.64)	10.76 (8.68-13.31)	0.064
2-h PG, mmol/L	18.93 (15.40-22.63)	18.31 (14.15-21.20)	18.58 (14.56-22.99)	18.75 (15.23-23.59)	19.93 (17.23-22.63)	0.276
Fasting insulin, µIU/mL	9.37 (6.38-13.36)	8.39 (4.46-11.48)	7.45 (4.78-10.16)	10.57 (7.92-14.64)	11.84 (8.86-18.88)	<0.001
Fasting C-peptide, ng/mL	1.95 (1.30-2.94)	1.47 (0.45-1.95)	1.81 (0.98-2.38)	2.81 (1.71-3.33)	3.06 (2.08-3.50)	<0.001
HOMA-IR	3.98 (2.17-6.16)	2.54 (1.63-4.93)	2.88 (1.87-4.74)	4.65 (3.31-6.35)	5.79 (4.59-9.17)	<0.001
ALT, U/L	22.4 (13.6-36.9)	14.8 (10.9-20.8)	21.9 (15.4-32.8)	26.5 (22.6-49.5)	36.9 (21.6-55.0)	<0.001
AST, U/L	19.3 (14.9-26.2)	16.0 (12.4-20.1)	18.5 (14.5-23.3)	22.0 (17.1-28.1)	27.6 (20.1-41.3)	<0.001
GGT, U/L	28.0 (18.0-47.0)	18.0 (13.0-25.5)	26.5 (17.8-39.8)	48.0 (31.0-79.5)	34.0 (28.0-56.5)	<0.001
Uric acid, µmol/L	293 (242-355)	288 (213-348)	259 (226-336)	298 (264-356)	330 (288-398)	0.003
TG, mmol/L	1.62 (1.01-2.52)	1.01 (0.77-1.69)	1.48 (1.06-1.99)	2.40 (1.47-3.69)	1.98 (1.66-3.63)	<0.001
TC, mmol/L	4.77 (4.03-5.67)	4.47 (3.71-5.52)	4.96 (4.46-5.47)	4.94 (4.20-5.74)	5.15 (4.07-6.30)	0.111
HDL-C, mmol/L	1.13 (0.99-1.32)	1.16 (1.04-1.44)	1.12 (0.96-1.31)	1.04 (0.92-1.21)	1.15 (0.99-1.24)	0.029
LDL-C, mmol/L	2.64 (1.98-3.38)	2.29 (1.84-3.10)	2.87 (2.31-3.25)	2.78 (2.02-3.25)	2.91 (2.22-3.91)	0.058
HbA1c, %	9.40 (8.40-11.10)	9.40 (8.30-11.00)	9.90 (8.47-11.40)	9.40 (7.95-11.30)	9.20 (8.45-10.35)	0.689
Male sex, n (%)	103 (67.3)	34 (66.7)	24 (66.7)	18 (66.7)	27 (69.2)	0.993
Antihypertensive therapy, n (%)	80 (52.3)	27 (52.9)	16 (44.4)	17 (63.0)	20 (51.3)	0.543
Statin therapy, n (%)	34 (22.2)	17 (33.3)	6 (16.7)	6 (22.2)	5 (12.8)	0.099
Glucose-lowering therapy, n (%)	126 (82.4)	40 (78.4)	28 (77.8)	21 (77.8)	37 (94.9)	0.086
Drinking history, n (%)	21 (13.7)	9 (17.6)	3 (8.3)	4 (14.8)	5 (12.8)	0.681
Smoking history, n (%)	52 (34.0)	20 (39.2)	13 (36.1)	9 (33.3)	10 (25.6)	0.591

Values are median (interquartile range) or n (%). Continuous variables were compared using the Kruskal–Wallis test; categorical variables were compared using Pearson χ² tests (df=3) when expected cell counts were adequate. Fisher–Freeman–Halton exact tests were used for glucose-lowering therapy and drinking history because of sparse expected counts.

UDFF grades: S0 <8%, S1 8% to <14%, S2 14% to <20%, and S3 ≥20%. HOMA-IR was treated as a descriptive metabolic variable and was not included in the primary multivariable models.

UDFF, ultrasound-derived fat fraction; CIMT, carotid intima-media thickness; BMI, body mass index; SBP, systolic blood pressure; DBP, diastolic blood pressure; FPG, fasting plasma glucose; 2-h PG, 2-hour postprandial glucose; HOMA-IR, homeostasis model assessment of insulin resistance; ALT, alanine aminotransferase; AST, aspartate aminotransferase; GGT, γ-glutamyl transferase; TG, triglycerides; TC, total cholesterol; HDL-C, high-density lipoprotein cholesterol; LDL-C, low-density lipoprotein cholesterol; HbA1c, glycated hemoglobin.

Continuous UDFF was the primary exposure. CIMT was analyzed using linear regression and prevalent carotid plaque using logistic regression. For both outcomes, an unadjusted model was fitted first, followed by Model 1 adjusted for age and sex and Model 2 additionally adjusted for statin therapy and LDL-C. Age and sex constituted the core demographic adjustment in Model 1, whereas Model 2 additionally incorporated LDL-C and statin therapy to account for lipid-related vascular risk and treatment. Potential residual confounding by other clinically distinct vascular-risk domains was evaluated in sensitivity analyses. The broader traditional vascular-risk model additionally included BMI, systolic blood pressure, smoking status, diabetes duration, and triglycerides. An expanded sensitivity model further incorporated HbA1c and antihypertensive therapy to account for glycemic control and blood-pressure treatment. BMI was used as the representative adiposity measure rather than simultaneously including BMI and waist circumference to avoid redundant adjustment for closely related anthropometric measures. Exclusion of participants with prior clinical ASCVD was evaluated in a separate sensitivity analysis.

Model assumptions, multicollinearity, influential observations, and functional form were assessed using standard regression diagnostics. Additional sensitivity analyses included log-transformed CIMT, HC3 heteroscedasticity-consistent standard errors, restricted cubic spline analysis, Firth penalized logistic regression, leave-one-out analysis, and 1,000 bootstrap resamples. Incremental statistical information provided by UDFF was assessed using changes in R² and nested-model F tests for CIMT and changes in C-statistic, AIC, likelihood-ratio tests, and DeLong tests for carotid plaque. Incremental analyses were evaluated in both the primary clinical adjustment model and the broader traditional vascular-risk model, because the magnitude of incremental discrimination may depend on the covariates included in the reference model. To compare UDFF with conventional qualitative liver ultrasonography, otherwise identical logistic regression models for prevalent carotid plaque were fitted by adding either continuous UDFF or the original four-category conventional ultrasonographic steatosis grade to the base clinical covariates (age, sex, statin therapy, and LDL-C). Conventional ultrasonographic grade was entered as a categorical variable, with absent steatosis as the reference category. Model fit was assessed using likelihood-ratio tests and information criteria, and discrimination was assessed using C-statistics with DeLong tests for paired comparisons. Alternative ordinal (0–3) and binary (absent vs any steatosis) codings of conventional ultrasonographic steatosis were examined in sensitivity analyses. Liver shear-wave velocity was compared across UDFF grades using the Kruskal-Wallis test, and its association with continuous UDFF was assessed using Spearman rank correlation. To evaluate potential confounding by liver stiffness, liver shear-wave velocity was additionally entered into the primary multivariable models as a sensitivity covariate. For logistic regression models, the events-per-variable (EPV) ratio was calculated to evaluate potential overfitting. In the primary plaque model including five predictors (UDFF, age, sex, statin therapy, and LDL-C), there were 89 plaque events and 64 non-events, corresponding to 17.8 events and 12.8 non-events per predictor, respectively. Statistical analyses were performed using IBM SPSS Statistics version 27.0 (IBM Corp., Armonk, NY, USA) and Stata version 18.0 (StataCorp LLC, College Station, TX, USA). All tests were two-sided, with P<0.05 considered statistically significant.

## 3 Results

### 3.1 Participant selection and baseline characteristics

A total of 173 potentially eligible patients with T2DM were screened. Fifteen did not meet the clinical eligibility criteria: 3 were younger than 18 years, 8 had viral hepatitis, and 4 had thyroid disease. Five additional patients were excluded because of incomplete key clinical or laboratory data. The final analytic cohort comprised 153 participants (**Figure 2**). All variables included in the primary analyses were complete in the final cohort, and no statistical imputation was performed (**Supplementary Table S1**).

**Figure 2:**
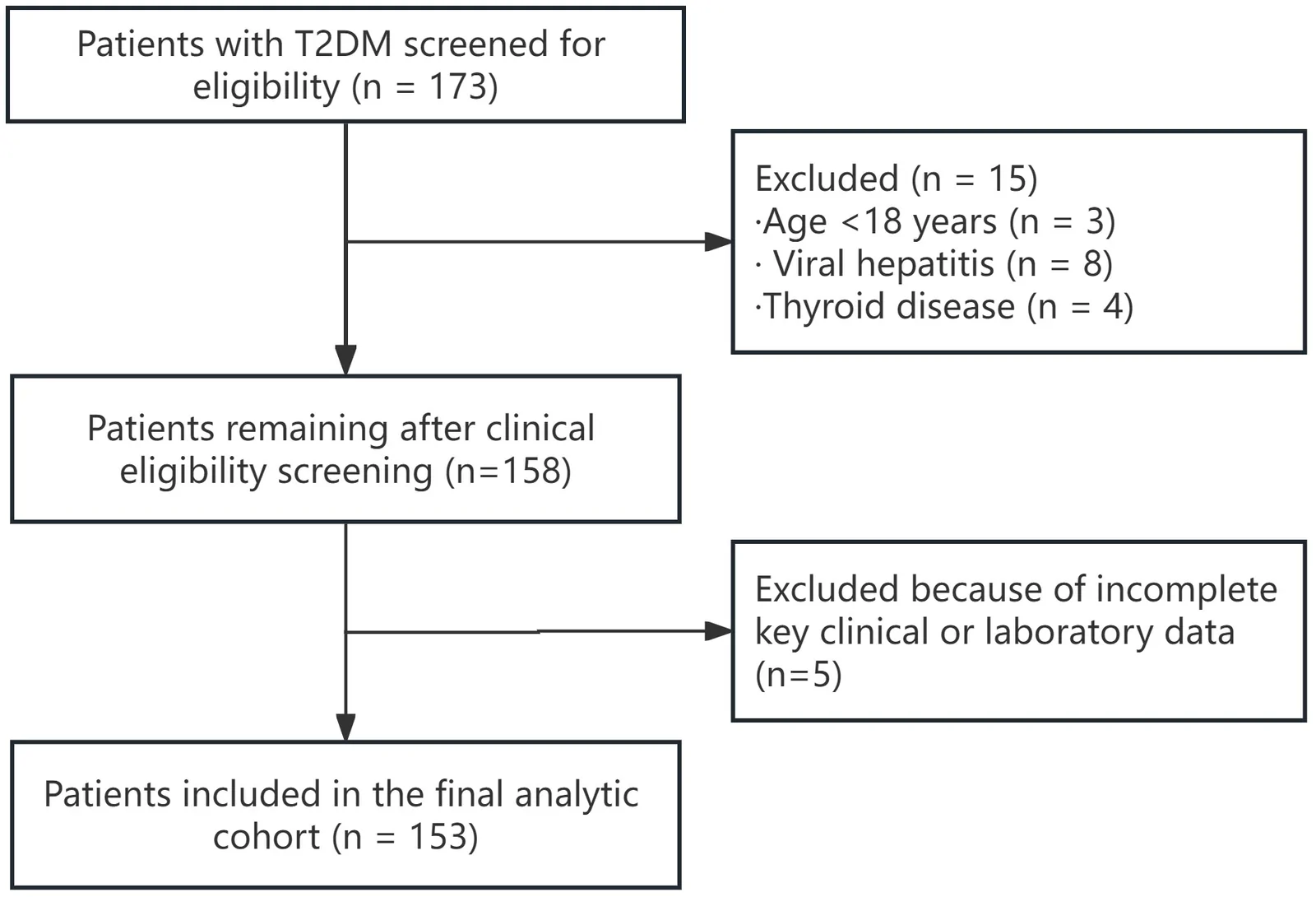
Flow diagram of participant selection.

The median age of the study population was 60 years (interquartile range [IQR], 51–69 years), and 103 participants (67.3%) were men. The median UDFF was 11% (IQR, 6%-20%). According to the UDFF categories, 51 participants were classified as S0, 36 as S1, 27 as S2, and 39 as S3. The median CIMT in the overall cohort was 0.85 mm (IQR, 0.74–1.05 mm). CIMT thickening was present in 54 of 153 participants (35.3%; exact 95% CI, 27.7%–43.4%), and carotid plaque was present in 89 of 153 participants (58.2%; exact 95% CI, 49.9%–66.1%). Twenty-eight participants (18.3%) had a history of clinical ASCVD.

Clinical, anthropometric, biochemical, and ultrasound characteristics according to UDFF grade are summarized in **Table 1**. Several anthropometric, metabolic, and hepatic variables differed significantly across UDFF grades, whereas waist-to-hip ratio did not differ significantly. After Benjamini-Hochberg FDR adjustment, the principal differences in anthropometric, insulin-related, hepatic, and triglyceride variables remained significant, whereas the marginal difference in HDL-C did not remain statistically significant (FDR-adjusted P = 0.055; **Supplementary Table S10**). Correlation analyses showed that continuous UDFF was associated with several anthropometric, insulin-related, hepatic, lipid, and vascular variables and was strongly correlated with conventional qualitative ultrasonographic steatosis grade (**Supplementary Table S2**). Median liver shear-wave velocity was 1.22 m/s (IQR, 1.04-1.35 m/s) in the overall cohort and did not differ significantly across UDFF grades (P = 0.274; **Table 1**). Continuous UDFF was not significantly correlated with liver shear-wave velocity (Spearman ρ=−0.114, P = 0.160; **Supplementary Table S2**).

### 3.2 CIMT and carotid plaque across UDFF grades

CIMT differed significantly across UDFF grades. Median CIMT values were 0.80 mm (IQR, 0.70-0.90 mm) in S0, 0.85 mm (IQR, 0.75-1.01 mm) in S1, 0.85 mm (IQR, 0.65-1.00 mm) in S2, and 1.10 mm (IQR, 0.95-1.20 mm) in S3. An ordered increase across UDFF categories was observed using the Jonckheere-Terpstra test (J = 6101, P for trend<0.001).

However, the pattern was not characterized by uniform pairwise increases between successive grades. Holm-adjusted Dunn *post hoc* comparisons showed that CIMT was significantly higher in S3 than in S0, S1, and S2 (all adjusted P<0.001), whereas no significant pairwise differences were observed among S0, S1, and S2. Thus, the categorical difference in CIMT was most pronounced in participants with S3 UDFF values (**Figure 3A**).

**Figure 3:**
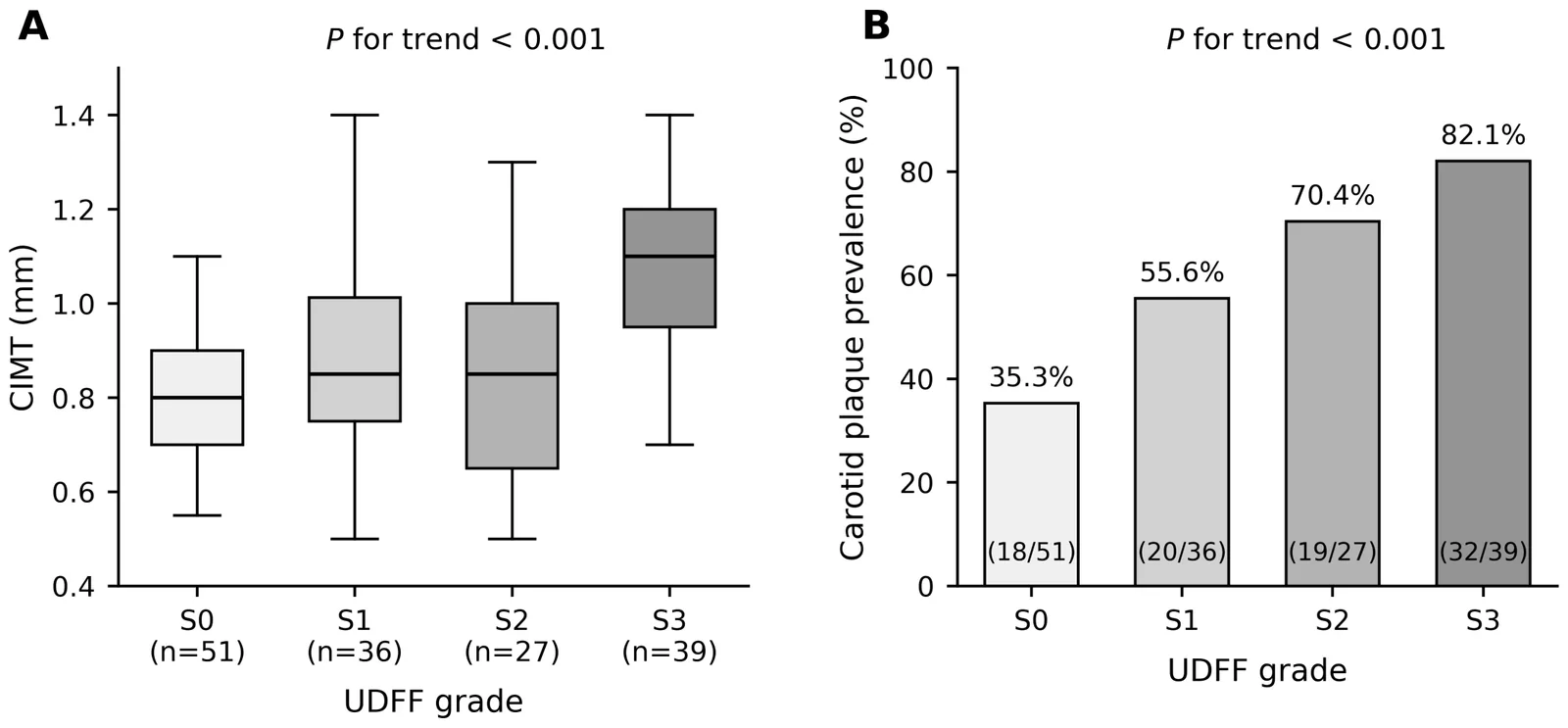
Carotid vascular findings across UDFF grades. **(A)** Distribution of carotid intima-media thickness (CIMT) across UDFF grades. CIMT showed a significant ordered trend across S0-S3 (Jonckheere-Terpstra J = 6101, P for trend<0.001), with the most pronounced difference observed in S3. **(B)** Prevalence of carotid plaque across UDFF grades. Plaque prevalence was 35.3%, 55.6%, 70.4%, and 82.1% in S0, S1, S2, and S3, respectively (Cochran-Armitage Z = 4.64, P for trend<0.001). UDFF, ultrasound-derived fat fraction; CIMT, carotid intima-media thickness.

The prevalence of carotid plaque increased across UDFF categories, from 35.3% (18/51) in S0 to 55.6% (20/36) in S1, 70.4% (19/27) in S2, and 82.1% (32/39) in S3. The Cochran-Armitage test demonstrated a significant ordered trend in plaque prevalence across UDFF grades (Z = 4.64, P for trend<0.001; **Figure 3B**). Detailed ordered-trend, *post hoc*, and exact confidence-interval results are provided in **Supplementary Table S3**. The categorical vascular pattern was also preserved after exclusion of participants within the dual-threshold indeterminate zones. For the ≥S1, ≥S2, and S3 boundaries, median CIMT remained significantly greater and plaque prevalence remained significantly higher in the respective rule-in than rule-out groups (all CIMT P<0.001; plaque P ≤ 0.001; **Supplementary Table S11**).

### 3.3 Association between UDFF and CIMT

Higher continuous UDFF was associated with greater CIMT in the unadjusted analysis and remained associated after multivariable adjustment. In Model 2, each 1-percentage-point increase in UDFF was associated with a 0.0105-mm higher CIMT (95% CI 0.0080-0.0131, P<0.001; **Table 2**). Sensitivity analyses using robust standard errors, log-transformed CIMT, and spline modeling yielded consistent findings.

**Table 2:** Association between continuous UDFF and CIMT.

Model	N	B, mm per 1%-point UDFF	Standardized β	95% CI	P value	R²	Adjusted R²
Unadjusted	153	0.0119	0.551	0.0090-0.0147	<0.001	0.304	0.299
Model 1: age + sex	153	0.0111	0.514	0.0085-0.0136	<0.001	0.473	0.463
Model 2: age + sex + statin therapy + LDL-C	153	0.0105	0.490	0.0080-0.0131	<0.001	0.498	0.481

B represents the change in CIMT (mm) associated with a 1-percentage-point increase in UDFF. Model 1 was adjusted for age and sex. Model 2 was additionally adjusted for statin therapy and LDL-C.

### 3.4 Association between UDFF and carotid plaque

Continuous UDFF was associated with prevalent carotid plaque in both unadjusted and multivariable analyses. In Model 2, each 1-percentage-point increase in UDFF was associated with higher odds of prevalent carotid plaque (OR = 1.109, 95% CI 1.056-1.164, P<0.001; **Table 3**). Firth penalized logistic regression yielded a similar estimate (OR = 1.102, 95% CI 1.055-1.160, P<0.001; **Supplementary Table S7**), and restricted cubic spline analysis showed no significant evidence of nonlinearity (P = 0.164; **Supplementary Figure S1**). The C-statistics for Model 1 and Model 2 were both 0.747 when rounded to three decimal places; the corresponding unrounded values were 0.7471 and 0.7474, respectively, and their difference was not statistically significant (paired DeLong P = 0.961).

**Table 3:** Association between continuous UDFF and prevalent carotid plaque.

Model	N	OR per 1%-point UDFF	95% CI	P value	AIC	C-statistic
Unadjusted	153	1.109	1.059-1.161	<0.001	185.73	0.734
Model 1: age + sex	153	1.111	1.060-1.166	<0.001	185.30	0.747
Model 2: age + sex + statin therapy + LDL-C	153	1.109	1.056-1.164	<0.001	189.01	0.747

OR represents the change in odds of prevalent carotid plaque associated with a 1-percentage-point increase in UDFF. Model 1 was adjusted for age and sex. Model 2 was additionally adjusted for statin therapy and LDL-C. The unrounded C-statistics were 0.7471 for Model 1 and 0.7474 for Model 2; the difference was not statistically significant by the paired DeLong test (P = 0.961). AIC, Akaike information criterion; OR, odds ratio; CI, confidence interval; UDFF, ultrasound-derived fat fraction.

### 3.5 Sensitivity analyses and model stability

In sensitivity analyses incorporating broader traditional vascular-risk factors or additional medication-use indicators, the associations between UDFF and CIMT or carotid plaque remained materially unchanged (**Supplementary Table S4**). In an expanded sensitivity model further incorporating HbA1c and antihypertensive therapy, UDFF remained associated with both CIMT (B = 0.0113 mm per 1-percentage-point increase, 95% CI 0.0082–0.0145, P<0.001) and prevalent carotid plaque (OR = 1.117, 95% CI 1.051–1.187, P<0.001; **Supplementary Table S4**). After exclusion of 28 participants with prior clinical ASCVD (n=125), the associations also remained similar for CIMT (B = 0.0102 mm, 95% CI 0.0072-0.0132, P<0.001) and carotid plaque (OR = 1.105, 95% CI 1.048-1.165, P<0.001; **Supplementary Table S5**). Model diagnostics, leave-one-out analyses, and bootstrap validation supported the stability of the primary models (**Supplementary Tables S6**, **S7**; **Supplementary Figure S5**). Additional adjustment for liver shear-wave velocity also yielded materially unchanged associations for CIMT (B = 0.0105 mm per 1-percentage-point increase in UDFF, 95% CI 0.0080-0.0131, P<0.001) and carotid plaque (OR = 1.108, 95% CI 1.055-1.163, P<0.001; **Supplementary Table S4**). The primary logistic regression model included five predictors, 89 plaque events, and 64 non-events, corresponding to 17.8 events and 12.8 non-events per predictor, respectively, and supporting an acceptable level of model complexity.

### 3.6 Incremental statistical information provided by UDFF

Adding UDFF to the primary clinical adjustment model increased R² for CIMT from 0.274 to 0.498 (ΔR²=0.224, P<0.001) and the C-statistic for prevalent carotid plaque from 0.633 to 0.747 (ΔC-statistic=0.114; DeLong P = 0.018; **Table 4**). After verification of the broader traditional vascular-risk analysis using the complete covariate set specified in the Statistical Analysis section, addition of UDFF increased the C-statistic from 0.697 to 0.758 (ΔC-statistic=0.061; DeLong P = 0.045; **Table 4**). The smaller increment observed in the broader model indicates that the magnitude of incremental discrimination was dependent on the covariates included in the reference model. These analyses assess within-cohort statistical discrimination and do not establish prospective predictive utility or clinical benefit.

**Table 4:** Incremental statistical information provided by UDFF.

Panel A. CIMT							
Model	N	R²	Adjusted R²	ΔR²	Nested F	P value	
Clinical covariates only	153	0.274	0.255	–	–	–	
Clinical covariates + UDFF	153	0.498	0.481	0.224	65.66	<0.001	
Panel B. Prevalent carotid plaque							
Model	N	AIC	C-statistic	ΔC-statistic	LR χ²	LR P	DeLong P vs base
Clinical covariates only	153	210.23	0.633	–	–	–	–
Clinical covariates + UDFF	153	189.01	0.747	0.114	23.23	<0.001	0.018
Panel C. Sensitivity analysis using a broader traditional vascular-risk model							
Model			N	C-statistic	ΔC-statistic	DeLong P	
Broader traditional vascular-risk covariates only			153	0.697	—	—	
Broader traditional vascular-risk covariates + UDFF			153	0.758	0.061	0.045	

Clinical covariates were age, sex, statin therapy, and LDL-C. For CIMT, nested linear models were compared using an F test. For prevalent carotid plaque, nested logistic models were compared using a likelihood-ratio (LR) test and C-statistics were compared using the DeLong test.

These analyses assess cross-sectional incremental statistical information and do not establish prospective risk prediction or clinical utility.

The broader traditional vascular-risk model included age, sex, statin therapy, LDL-C, BMI, systolic blood pressure, smoking status, diabetes duration, and triglycerides.

### 3.7 Comparison with conventional qualitative liver ultrasonography

Conventional ultrasonographic steatosis grade was strongly correlated with UDFF (ρ=0.750, P<0.001). In otherwise identical clinical models, the C-statistic was 0.657 after adding the original four-category conventional ultrasonographic steatosis grade and 0.747 after adding continuous UDFF (Δ=0.090, DeLong P = 0.008). The overall contribution of the four-category conventional ultrasonographic grade to the base clinical model was not statistically significant (likelihood-ratio χ²=2.96, df=3, P = 0.399), whereas addition of UDFF significantly improved model fit (likelihood-ratio χ²=23.23, df=1, P<0.001). Adding the four-category conventional ultrasonographic grade to the UDFF model increased the C-statistic from 0.747 to 0.787, although the increase in discrimination was not statistically significant (DeLong P = 0.141). Alternative ordinal and binary codings of conventional ultrasonographic steatosis yielded materially similar conclusions (**Supplementary Table S8**; **Supplementary Figure S4**).

### 3.8 Inter-reader reanalysis agreement

In the 30-examination reanalysis subset, UDFF and CIMT demonstrated high and good inter-reader reanalysis agreement, respectively. The ICC was 0.933 (95% CI, 0.864–0.968) for UDFF and 0.830 (95% CI, 0.672–0.915) for CIMT (**Supplementary Table S9**). Bland–Altman analyses are presented in **Supplementary Figures S2** and **S3**. These estimates quantify agreement between two readers who reanalyzed the same stored examinations and should not be interpreted as interoperator reproducibility involving independent image acquisition.

## 4 Discussion

In this exploratory cross-sectional study of patients with T2DM, higher UDFF values were associated with greater CIMT and prevalent carotid plaque. The associations were similar after multivariable adjustment, in broader sensitivity models, and after exclusion of participants with prior clinical ASCVD. CIMT also differed across UDFF grades, although the difference was mainly attributable to the S3 group rather than to a uniform stepwise increase from S0 to S3.

UDFF was correlated with measures of adiposity, fasting C-peptide, HOMA-IR, triglycerides, liver enzymes, and conventional ultrasonographic steatosis grade. This pattern is consistent with the clustering of adiposity, insulin resistance, dyslipidemia, and hepatic steatosis in T2DM (29, 30). The insulin-related findings require caution because fasting insulin and HOMA-IR may be influenced by glucose-lowering treatment, while aminotransferase levels are not specific markers of hepatic steatosis. Previous prospective studies have reported good agreement between UDFF and MRI-PDFF (15, 16, 25), but the absence of MRI-PDFF or histology in our cohort precluded direct reference-standard validation.

Previous studies have generally linked hepatic steatosis with greater CIMT and carotid plaque (19, 31–33), but findings in patients with T2DM have not been consistent. Kim et al. (34) reported greater CIMT only in patients with T2DM and insulin resistance, whereas Petit et al. (35) found no association between fatty liver and CIMT in a T2DM cohort. In a large Chinese inpatient T2DM cohort, Guo et al. found that NAFLD was associated with carotid plaque but not with increased CIMT after multivariable adjustment (36). Differences in the definition of hepatic steatosis, metabolic characteristics of the study populations, and covariate adjustment may partly explain these inconsistent findings. In our cohort, plaque prevalence increased across UDFF grades, whereas the categorical difference in CIMT was mainly confined to S3 and should therefore be interpreted as exploratory. Because the S1 and S2 groups were relatively small and spline analysis did not show significant nonlinearity, this pattern does not establish a discrete UDFF threshold for carotid vascular abnormalities.

The incremental findings depended on the clinical variables included in the comparator model. Adding UDFF to the primary clinical adjustment model increased the C-statistic for prevalent carotid plaque from 0.633 to 0.747 (ΔC-statistic=0.114). When a broader set of traditional vascular-risk factors was included, the corresponding increase was smaller, from 0.697 to 0.758 (ΔC-statistic=0.061; DeLong P = 0.045). Thus, although UDFF provided incremental statistical information in both models, the attenuation of the C-statistic increment in the broader model indicates that the magnitude of incremental discrimination was dependent on the information already contained in the reference model. Accordingly, the observed improvement should not be interpreted as a model-independent property of UDFF or as evidence of established prospective predictive utility. These findings suggest that the two ultrasound measures may contain partially overlapping but not identical statistical information. Both measurements were obtained during routine clinical examinations, and the results should be viewed as within-cohort comparisons rather than evidence that UDFF is universally superior to qualitative ultrasonography. Bootstrap validation also indicated some optimism in model performance: the apparent C-statistic of 0.747 decreased to 0.716 after optimism correction. Finally, the inter-reader analysis assessed repeated interpretation and ROI placement on the same stored acquisitions and did not include variability from independent image acquisition.

One possible link between hepatic fat and carotid vascular abnormalities is the metabolic disturbance shared by the liver, adipose tissue, and arterial wall (37). Hepatic and adipose-tissue insulin resistance can increase fatty-acid delivery to the liver and promote the secretion of triglyceride-rich VLDL particles, resulting in a more atherogenic lipid profile (2, 29, 38). The correlations of UDFF with HOMA-IR and triglycerides in our cohort are compatible with this pathway, but they do not establish mediation. Inflammatory, endothelial, and prothrombotic markers were not measured. Hepatic fibrosis may also be more closely related to cardiovascular abnormalities than steatosis alone (39). In the present study, additional adjustment for liver shear-wave velocity did not materially alter the associations of UDFF with either CIMT or carotid plaque. However, shear-wave velocity is a fibrosis-related imaging measure rather than a reference-standard fibrosis stage (40); therefore, residual confounding by hepatic fibrosis cannot be completely excluded.

Because the liver and carotid measurements were obtained within the same 24-hour period, the cross-sectional design cannot establish temporal ordering or causal direction. The observed associations may reflect metabolic factors shared by hepatic steatosis and carotid disease rather than a direct effect of hepatic fat on the arterial wall. Reverse causation also cannot be excluded: carotid atherosclerosis and its accompanying systemic vascular or hemodynamic alterations could, in principle, influence hepatic perfusion or lipid metabolism, although this possibility cannot be tested in the present dataset. Medication-related reverse causation is also possible, because patients with longer diabetes duration or greater vascular risk may be more likely to receive statins or glucose-lowering therapies that subsequently modify lipid metabolism, insulin sensitivity, and hepatic fat accumulation. Although statin use was included in the adjusted models, detailed information on treatment indication, drug class, dose, duration, adherence, and cumulative exposure was incomplete. Longitudinal studies with repeated liver and carotid measurements and detailed treatment exposure are needed to clarify temporal direction and causal relationships (41).

Several limitations should also be acknowledged. First, participants were retrospectively identified from an inpatient endocrinology population. This inpatient case mix represents a relatively high-risk and clinically complex T2DM population and may limit the generalizability of our findings to ambulatory or community-based patients with better-controlled diabetes or to populations undergoing opportunistic screening. Exclusion of five patients with incomplete clinical or laboratory records may have introduced selection bias. Second, this was a single-center study with a modest sample size. The primary models had acceptable event-to-parameter ratios, but the modest sample size limited the precision of subgroup analyses and model-performance estimates. Third, MRI-PDFF and liver histology were unavailable. Although liver shear-wave velocity was available as a supplementary fibrosis-related imaging measure, reference-standard fibrosis staging was not available. Fourth, although the associations remained materially unchanged after broader adjustment for adiposity, blood pressure, smoking, diabetes duration, lipid, glycemic, and treatment-related factors, residual confounding remains possible, and detailed information on medication indication, dose, duration, adherence, and cumulative exposure was incomplete. Fifth, carotid plaque was analyzed only as a binary outcome because standardized measures of plaque burden were unavailable. Although a mean CIMT ≥1.0 mm was retained for descriptive and exploratory comparison with previous studies, this threshold does not account for age-, sex-, population-, or acquisition-related variation. Accordingly, the categorical CIMT-thickening findings should be interpreted cautiously, and the main conclusions are based on continuous CIMT. Finally, the reproducibility analysis was limited to inter-reader reanalysis of the same stored examinations. It therefore did not capture variability related to independent image acquisition. Formal intra-reader reproducibility and inter-reader agreement for carotid plaque classification were also unavailable.

## 5 Conclusion

In this exploratory cross-sectional study, higher UDFF values were associated with greater continuous CIMT and prevalent carotid plaque in patients with T2DM, with similar findings across sensitivity analyses. UDFF provided incremental statistical information within this cohort, although the magnitude of improvement in plaque discrimination was smaller in the broader traditional vascular-risk model than in the primary clinical adjustment model. Given the cross-sectional design and absence of MRI-PDFF, histologic validation, and reference-standard fibrosis staging, prospective multicenter studies are needed to determine the clinical significance and external validity of these findings.

## Data Availability

The datasets presented in this article are not readily available because the datasets generated and/or analyzed during the current study are not publicly available due to the protection of patient privacy but are available from the corresponding author on reasonable request. Requests to access the datasets should be directed to ujs_zhangxin@163.com.
