# Supplementary material for: Association of ultrasound-derived fat fraction with carotid intima-media thickness and carotid plaque in patients with type 2 diabetes: an exploratory cross-sectional study
Source: Front Endocrinol (Lausanne). 2026 Sep 25;17:1940570. doi: 10.3389/fendo.2026.1940570 (PMC13649457; doi:10.3389/fendo.2026.1940570)
Supplement: Supplementary file 1 [file Table1.docx]

**Supplementary Table S1. Missing-data assessment in the final analytic cohort**

| **Variable** | **Expected N** | **Missing n** | **Missing %** |
| --- | --- | --- | --- |
| UDFF | 153 | 0 | 0.0 |
| UDFF grade | 153 | 0 | 0.0 |
| Liver shear-wave velocity | 153 | 0 | 0.0 |
| Conventional qualitative US grade | 153 | 0 | 0.0 |
| CIMT | 153 | 0 | 0.0 |
| Carotid plaque | 153 | 0 | 0.0 |
| Age | 153 | 0 | 0.0 |
| Sex | 153 | 0 | 0.0 |
| BMI | 153 | 0 | 0.0 |
| Waist circumference | 153 | 0 | 0.0 |
| Hip circumference | 153 | 0 | 0.0 |
| Waist-to-hip ratio | 153 | 0 | 0.0 |
| SBP | 153 | 0 | 0.0 |
| DBP | 153 | 0 | 0.0 |
| Antihypertensive therapy | 153 | 0 | 0.0 |
| Statin therapy | 153 | 0 | 0.0 |
| Glucose-lowering therapy | 153 | 0 | 0.0 |
| Smoking history | 153 | 0 | 0.0 |
| Drinking history | 153 | 0 | 0.0 |
| Diabetes duration | 153 | 0 | 0.0 |
| FPG | 153 | 0 | 0.0 |
| 2-h PG | 153 | 0 | 0.0 |
| Fasting insulin | 153 | 0 | 0.0 |
| Fasting C-peptide | 153 | 0 | 0.0 |
| HOMA-IR | 153 | 0 | 0.0 |
| ALT | 153 | 0 | 0.0 |
| AST | 153 | 0 | 0.0 |
| GGT | 153 | 0 | 0.0 |
| Uric acid | 153 | 0 | 0.0 |
| TG | 153 | 0 | 0.0 |
| TC | 153 | 0 | 0.0 |
| HDL-C | 153 | 0 | 0.0 |
| LDL-C | 153 | 0 | 0.0 |
| HbA1c | 153 | 0 | 0.0 |
| Prior clinical ASCVD variables | 153 | 0 | 0.0 |

Five otherwise clinically eligible patients were excluded before construction of the final analytic cohort because of substantial missing key clinical and laboratory data. Multiple variables were missing in some individuals; therefore, missingness categories were not mutually exclusive. No missing values remained among the 153 participants included in the final analytic cohort, and no statistical imputation was performed.

**Supplementary Table S2. Spearman correlations of continuous UDFF with metabolic, hepatic, and vascular variables**

| **Variable** | **Spearman ρ** | **P value** |
| --- | --- | --- |
| BMI, kg/m² | 0.418 | <0.001 |
| Waist circumference, cm | 0.387 | <0.001 |
| Hip circumference, cm | 0.309 | <0.001 |
| Waist-to-hip ratio | 0.213 | 0.008 |
| FPG, mmol/L | 0.226 | 0.005 |
| Fasting insulin, µIU/mL | 0.300 | <0.001 |
| Fasting C-peptide, ng/mL | 0.612 | <0.001 |
| HOMA-IR | 0.392 | <0.001 |
| ALT, U/L | 0.502 | <0.001 |
| AST, U/L | 0.443 | <0.001 |
| GGT, U/L | 0.487 | <0.001 |
| Uric acid, µmol/L | 0.288 | <0.001 |
| TG, mmol/L | 0.477 | <0.001 |
| TC, mmol/L | 0.199 | 0.014 |
| HDL-C, mmol/L | -0.193 | 0.017 |
| LDL-C, mmol/L | 0.200 | 0.013 |
| HbA1c, % | -0.048 | 0.555 |
| CIMT, mm | 0.485 | <0.001 |
| Conventional qualitative US grade | 0.750 | <0.001 |
| Liver shear-wave velocity, m/s | -0.114 | 0.160 |

Conventional qualitative ultrasound grade was coded as an ordered variable (absent, mild, moderate, severe).

**Supplementary Table S3. Ordered-trend and post hoc analyses across UDFF grades**

**Panel A. Ordered trends**

| **Outcome** | **S0** | **S1** | **S2** | **S3** | **Test statistic** | **P value** |
| --- | --- | --- | --- | --- | --- | --- |
| CIMT, mm | 0.80 | 0.85 | 0.85 | 1.10 | J=6101 | <0.001 |
| Carotid plaque, n (%) | 18/51 (35.3) | 20/36 (55.6) | 19/27 (70.4) | 32/39 (82.1) | Z=4.64 | <0.001 |

**Panel B. Dunn post hoc comparisons for CIMT**

| **Pairwise comparison** | **Z** | **Raw P** | **Holm-adjusted P** |
| --- | --- | --- | --- |
| S0 vs S1 | -1.466 | 0.143 | 0.428 |
| S0 vs S2 | -1.003 | 0.316 | 0.632 |
| S0 vs S3 | -6.413 | <0.001 | <0.001 |
| S1 vs S2 | 0.316 | 0.752 | 0.752 |
| S1 vs S3 | -4.521 | <0.001 | <0.001 |
| S2 vs S3 | -4.495 | <0.001 | <0.001 |

**Panel C. Carotid plaque prevalence with exact 95% confidence intervals**

| **UDFF grade** | **N** | **Plaque n (%)** | **Exact 95% CI** |
| --- | --- | --- | --- |
| S0 | 51 | 18 (35.3) | 22.4-49.9 |
| S1 | 36 | 20 (55.6) | 38.1-72.1 |
| S2 | 27 | 19 (70.4) | 49.8-86.2 |
| S3 | 39 | 32 (82.1) | 66.5-92.5 |
| Overall | 153 | 89 (58.2) | 49.9–66.1 |

**Panel D. CIMT-thickening prevalence with exact 95% confidence intervals**

| **UDFF grade** | **N** | **CIMT thickening, n (%)** | **Exact 95% CI** |
| --- | --- | --- | --- |
| S0 | 51 | 7 (13.7) | 5.7–26.3 |
| S1 | 36 | 10 (27.8) | 14.2–45.2 |
| S2 | 27 | 9 (33.3) | 16.5–54.0 |
| S3 | 39 | 28 (71.8) | 55.1–85.0 |
| Overall | 153 | 54 (35.3) | 27.7–43.4 |

The 4×2 Fisher–Freeman–Halton exact omnibus test for CIMT-thickening prevalence across UDFF grades was P<0.001.

The 4×2 Fisher-Freeman-Halton exact omnibus test for plaque prevalence across UDFF grades was P<0.001.

Plaque prevalence was 35.3% (exact 95% CI, 22.4%–49.9%), 55.6% (38.1%–72.1%), 70.4% (49.8%–86.2%), and 82.1% (66.5%–92.5%) in S0, S1, S2, and S3, respectively.

**Supplementary Table S4. Sensitivity analyses for the association between UDFF and carotid vascular outcomes**

| **Model** | **N** | **Parameters** | **CIMT B (95% CI)** | **P value** | **Carotid plaque OR (95% CI)** | **P value** |
| --- | --- | --- | --- | --- | --- | --- |
| Primary model | 153 | 5 | 0.0105 (0.0080-0.0131) | <0.001 | 1.109 (1.056-1.164) | <0.001 |
| Traditional vascular-risk model | 153 | 10 | 0.0113 (0.0081-0.0145) | <0.001 | 1.115 (1.051-1.184) | <0.001 |
| Expanded vascular-risk model | 153 | 12 | 0.0113 (0.0082-0.0145) | <0.001 | 1.117 (1.051-1.187) | <0.001 |
| Medication-adjusted model | 153 | 7 | 0.0107 (0.0081-0.0134) | <0.001 | 1.105 (1.051-1.161) | <0.001 |
| Liver shear-wave velocity-adjusted model | 153 | 6 | 0.0105 (0.0080-0.0131) | <0.001 | 1.108 (1.055-1.163) | <0.001 |
| Fully adjusted Firth logistic model (plaque only) | 153 | 12 | - | - | 1.104 (1.047-1.174) | <0.001 |

Primary model: adjusted for age, sex, statin therapy, and LDL-C.

Traditional vascular-risk model: additionally adjusted for BMI, systolic blood pressure, smoking status, diabetes duration, and triglycerides.

Expanded vascular-risk model: adjusted for age, sex, statin therapy, LDL-C, BMI, systolic blood pressure, smoking status, diabetes duration, triglycerides, HbA1c, and antihypertensive therapy.

Medication-adjusted model: additionally adjusted for antihypertensive therapy and glucose-lowering therapy.

Liver shear-wave velocity-adjusted model: additionally adjusted for liver shear-wave velocity (median Vs).

Fully adjusted Firth logistic model: adjusted for age, sex, BMI, systolic blood pressure, smoking status, diabetes duration, LDL-C, triglycerides, statin therapy, antihypertensive therapy, and glucose-lowering therapy; Firth penalized logistic regression was applied for carotid plaque analysis.

**Supplementary Table S5. Sensitivity analysis excluding participants with prior clinical ASCVD**

**Panel A. CIMT**

| **Model** | **N** | **B, mm per 1%-point UDFF** | **Standardized β** | **95% CI** | **P value** |
| --- | --- | --- | --- | --- | --- |
| Unadjusted | 125 | 0.0115 | 0.517 | 0.0081-0.0149 | <0.001 |
| Model 1 | 125 | 0.0112 | 0.504 | 0.0082-0.0142 | <0.001 |
| Model 2 | 125 | 0.0102 | 0.458 | 0.0072-0.0132 | <0.001 |

**Panel B. Prevalent carotid plaque**

| **Model** | **N** | **Plaque events/non-events** | **OR per 1%-point UDFF** | **95% CI** | **P value** |
| --- | --- | --- | --- | --- | --- |
| Unadjusted | 125 | 72/53 | 1.104 | 1.052-1.160 | <0.001 |
| Model 1 | 125 | 72/53 | 1.109 | 1.053-1.168 | <0.001 |
| Model 2 | 125 | 72/53 | 1.105 | 1.048-1.165 | <0.001 |
| Firth Model 2 | 125 | 72/53 | 1.097 | 1.047-1.159 | <0.001 |

Twenty-eight participants with prior clinical atherosclerotic cardiovascular disease (ASCVD) were excluded. Clinical ASCVD was defined as a documented history of coronary artery disease, myocardial infarction, ischemic stroke or transient ischemic attack, peripheral arterial disease, or prior coronary or carotid revascularization; overlapping conditions were permitted. Model 1 was adjusted for age and sex. Model 2 was additionally adjusted for statin therapy and LDL-C. Firth Model 2 applied Firth penalized logistic regression using the same covariates as Model 2.

**Supplementary Table S6. Diagnostic and sensitivity analyses for the primary CIMT Model 2**

| **Diagnostic / sensitivity analysis** | **Result** | **Interpretation** |
| --- | --- | --- |
| Residual Shapiro-Wilk | W=0.991; P=0.409 | Residual normality not rejected |
| Breusch-Pagan test | P=0.593 | No evidence of heteroscedasticity |
| White test | P=0.810 | No evidence of heteroscedasticity |
| RESET | P=0.585 | No strong evidence of functional-form misspecification |
| Variance inflation factors | UDFF=1.070; age=1.051; sex=1.038; statin therapy=1.148; LDL-C=1.166 | All VIFs <1.2; no material multicollinearity |
| Spline nonlinearity | P=0.120 | No strong evidence of nonlinearity |
| HC3 robust estimate | B=0.01053; 95% CI 0.00795-0.01310 | Consistent with primary OLS estimate |
| Log (CIMT) sensitivity | B=0.01126; 95% CI 0.00826-0.01426; P<0.001 | Consistent direction and statistical significance |

Model 2 included UDFF, age, sex, statin therapy, and LDL-C.

**Supplementary Table S7. Diagnostic and internal-validation analyses for the primary carotid plaque Model 2**

**Panel A. Logistic-regression diagnostics**

| **Diagnostic** | **Result** | **Interpretation** |
| --- | --- | --- |
| MLE convergence | Converged | No convergence problem |
| VIF range | 1.038-1.166 | No material multicollinearity |
| Maximum Cook's distance | 0.064 | No Cook’s D >1 |
| Maximum leverage | 0.096 | No evidence that leverage materially altered the result |
| Maximum \|studentized residual\| | 3.917 | One residual outlier; leave-one-out analysis remained stable |
| Maximum \|deviance residual\| | 2.354 | No major deviance residual >3 |
| Hosmer-Lemeshow test | χ²=4.80; P=0.779 | No substantial lack of calibration detected |
| Leave-one-out UDFF OR range | 1.106-1.121 | Association was not driven by a single participant |
| Separation | None detected | Finite estimates and normal convergence |
| RCS overall association | P<0.001 | Overall association detected |
| RCS nonlinearity | P=0.164 | No strong evidence of nonlinearity |

**Panel B. Bootstrap internal validation**

| **Metric** | **Result** |
| --- | --- |
| Bootstrap resamples requested | 1,000 |
| Successful model fits | 1,000 |
| Apparent C-statistic | 0.747 |
| Mean bootstrap optimism | 0.032 |
| Optimism-corrected C-statistic | 0.716 |
| Bootstrap median UDFF OR | 1.114 |
| Bootstrap 95% percentile interval for UDFF OR | 1.064-1.181 |
| Median calibration slope | 0.836 |
| Calibration slope 95% percentile interval | 0.561-1.249 |

Each bootstrap sample refitted the complete Model 2 (UDFF + age + sex + statin therapy + LDL-C).

**Panel C. Firth penalized logistic regression sensitivity analysis**

| **Model** | **N** | **OR per 1%-point UDFF** | **95% CI** | **P value** |
| --- | --- | --- | --- | --- |
| Primary Model 2, standard logistic regression | 153 | 1.109 | 1.056–1.164 | <0.001 |
| Primary Model 2, Firth penalized logistic regression | 153 | 1.102 | 1.055–1.160 | <0.001 |

Both models included UDFF, age, sex, statin therapy, and LDL-C. Firth penalized logistic regression was performed as a sensitivity analysis to assess the robustness of the primary logistic-regression estimate.

**Supplementary Table S8. Comparison of conventional qualitative liver ultrasonography and UDFF for prevalent carotid plaque**

**Panel A. Model performance**

| **Model** | **N** | **AIC** | **BIC** | **C-statistic** |
| --- | --- | --- | --- | --- |
| Base clinical model | 153 | 210.23 | 225.38 | 0.633 |
| Base + conventional US four-category grade | 153 | 213.28 | 237.52 | 0.657 |
| Base + UDFF | 153 | 189.01 | 207.19 | 0.747 |
| Base + conventional US four-category grade + UDFF | 153 | 182.81 | 210.08 | 0.787 |

**Panel B. Nested likelihood-ratio comparisons**

| **Comparison** | **LR χ²** | **df** | **P** |
| --- | --- | --- | --- |
| Base → four-category conventional US | 2.96 | 3 | 0.399 |
| Base → UDFF | 23.23 | 1 | <0.001 |
| Four-category conventional US → combined | 32.47 | 1 | <0.001 |
| UDFF → combined | 12.20 | 3 | 0.007 |

**Panel C. C-statistic comparisons**

| **Comparison** | **ΔC-statistic** | **DeLong P** |
| --- | --- | --- |
| UDFF vs four-category conventional US | 0.090 | 0.008 |
| Combined vs four-category conventional US | 0.130 | 0.002 |
| Combined vs UDFF | 0.039 | 0.141 |

**Panel D. Sensitivity analyses using alternative codings of conventional ultrasonographic steatosis**

| **Alternative coding** | **Adjusted association** | **C-statistic** | **Comparison with UDFF** |
| --- | --- | --- | --- |
| Ordinal grade (0–3), per one-grade increase | OR=1.216 (95% CI 0.894–1.653); P=0.212 | 0.646 | ΔC-statistic=0.101; DeLong P=0.003 |
| Binary grade (absent vs any steatosis) | OR=1.821 (95% CI 0.893–3.712); P=0.099 | 0.657 | ΔC-statistic=0.091; DeLong P=0.007 |

The base clinical model included age, sex, statin therapy, and LDL-C. The combined model additionally included continuous UDFF and the four-category conventional ultrasonographic steatosis grade.

Ordinal analysis treated conventional ultrasonographic steatosis grade as an ordered variable coded 0–3 (absent, mild, moderate, and severe), with the reported OR representing the change per one-grade increase. Binary analysis dichotomized conventional ultrasonographic steatosis as absent versus any steatosis. Both models were adjusted for age, sex, statin therapy, and LDL-C. C-statistics from the alternative conventional ultrasonography models were compared with that of the corresponding UDFF model using the DeLong test.

The conventional ultrasonographic steatosis grades comprised 53 participants with absent, 45 with mild, 25 with moderate, and 30 with severe steatosis.

**Supplementary Table S9. Inter-reader reanalysis agreement based on the same stored examinations**

| **Measure** | **N** | **Reliability statistic** | **Estimate** | **95% CI** | **Mean difference (Reader 1 -Reader 2)** | **95% limits of agreement** | **CV (%)** |
| --- | --- | --- | --- | --- | --- | --- | --- |
| UDFF | 30 | ICC(2,1)/ICC(A,1) | 0.933 | 0.864-0.968 | -0.03 percentage points | -5.44 to 5.38 percentage points | 16.56 |
| CIMT | 30 | ICC(2,1)/ICC(A,1) | 0.830 | 0.672-0.915 | ≈0.00 mm | -0.188 to 0.188 mm | 7.82 |

ICCs were calculated using a two-way random-effects, absolute-agreement, single-measurement model. Both readers evaluated data obtained during the same original clinical examination. For UDFF, the second reader independently repositioned the region of interest and repeated the measurements on the five stored acquisitions, after which the median was calculated as the participant-level value. For CIMT, the second reader repeated the measurements on the same stored carotid images. Therefore, these results represent inter-reader reanalysis agreement rather than interoperator acquisition reproducibility. Plaque agreement was not calculated because the archived carotid examinations did not consistently contain complete standardized sweeps of all carotid segments required to establish plaque absence independently.

The within-subject coefficient of variation (CV) was calculated as the within-subject standard deviation divided by the overall mean and multiplied by 100%, where the within-subject standard deviation was estimated as the standard deviation of the paired reader differences divided by √2.

**Supplementary Table S10.** **Benjamini-Hochberg false-discovery-rate adjustment for exploratory between-grade comparisons in Table 1**

| **Variable** | **Unadjusted P** | **BH-FDR adjusted P** |
| --- | --- | --- |
| CIMT | <0.001 | <0.001 |
| CIMT thickening | <0.001 | <0.001 |
| Liver shear-wave velocity | 0.274 | 0.354 |
| Age | 0.353 | 0.435 |
| BMI | <0.001 | <0.001 |
| Waist circumference | <0.001 | <0.001 |
| Hip circumference | <0.001 | 0.001 |
| WHR | 0.081 | 0.129 |
| SBP | 0.818 | 0.844 |
| DBP | 0.003 | 0.005 |
| Diabetes duration | <0.001 | <0.001 |
| FPG | 0.064 | 0.108 |
| 2-h PG | 0.276 | 0.354 |
| Fasting insulin | <0.001 | <0.001 |
| Fasting C-peptide | <0.001 | <0.001 |
| HOMA-IR | <0.001 | <0.001 |
| ALT | <0.001 | <0.001 |
| AST | <0.001 | <0.001 |
| GGT | <0.001 | <0.001 |
| Uric acid | 0.003 | 0.005 |
| TG | <0.001 | <0.001 |
| TC | 0.111 | 0.154 |
| HDL-C | 0.029 | 0.055 |
| LDL-C | 0.058 | 0.103 |
| HbA1c | 0.689 | 0.734 |
| Male sex | 0.993 | 0.993 |
| Antihypertensive therapy | 0.543 | 0.643 |
| Statin therapy | 0.099 | 0.144 |
| Glucose-lowering therapy | 0.086 | 0.132 |
| Drinking history | 0.681 | 0.734 |
| Smoking history | 0.591 | 0.676 |
| Carotid plaque | <0.001 | <0.001 |

Benjamini-Hochberg FDR adjustment was applied to 32 exploratory between-grade comparisons in Table 1, including continuous CIMT, CIMT thickening, and carotid plaque. UDFF itself was excluded from the FDR family because UDFF grade was defined according to UDFF values.

**Supplementary Table S11. Grey-zone sensitivity analyses using the dual UDFF rule-out and rule-in thresholds**

| **Boundary** | **Excluded grey zone** | **Rule-out group** | **Rule-in group** | **CIMT, median (IQR), mm** | **Plaque prevalence** |
| --- | --- | --- | --- | --- | --- |
| ≥S1 | >6 to ≤10%, n=25 (16.3%) | UDFF ≤6%, n=45 | UDFF >10%, n=83 | 0.80 (0.70-0.90) vs 0.95 (0.80-1.10), P<0.001 | 28.9% vs 68.7%, P<0.001 |
| ≥S2 | >12 to ≤18%, n=30 (19.6%) | UDFF ≤12%, n=82 | UDFF >18%, n=41 | 0.80 (0.70-0.95) vs 1.10 (0.95-1.20), P<0.001 | 46.3% vs 78.0%, P=0.001 |
| S3 | >16 to ≤22%, n=22 (14.4%) | UDFF ≤16%, n=100 | UDFF >22%, n=31 | 0.80 (0.70-0.95) vs 1.10 (1.03-1.20), P<0.001 | 45.0% vs 90.3%, P<0.001 |

**Supplementary Figure S1. Restricted cubic spline for the association between UDFF and prevalent carotid plaque.**

Adjusted for age, sex, statin therapy, and LDL-C. Four knots were placed at the 5th, 35th, 65th, and 95th percentiles of UDFF (3%, 8%, 16%, and 33%). UDFF=11% was used as the reference. P for overall association<0.001; P for nonlinearity=0.164.

**Supplementary Figure S2. Bland–Altman plot of inter-reader reanalysis agreement for UDFF.**

Thirty examinations were randomly selected from the stored imaging dataset. The second reader independently repositioned the region of interest and repeated the measurements on the same five stored UDFF acquisitions obtained during the original clinical examination. No repeat ultrasound acquisition was performed. The mean difference between reader 1 and reader 2 was −0.03 percentage points, and the 95% limits of agreement were −5.44 to 5.38 percentage points.

**Supplementary Figure S3. Bland–Altman plot of inter-reader reanalysis agreement for CIMT.**

Thirty examinations were randomly selected from the stored imaging dataset. The second reader independently repeated the CIMT measurements on the same stored carotid ultrasound images obtained during the original clinical examination. No repeat carotid ultrasound acquisition was performed. The mean difference between reader 1 and reader 2 was approximately 0.00 mm, and the 95% limits of agreement were -0.188 to 0.188 mm.

**Supplementary Figure S4. Receiver-operating-characteristic curves comparing conventional qualitative liver ultrasonography and UDFF for prevalent carotid plaque.**

Receiver-operating-characteristic curves comparing the original four-category conventional ultrasonographic steatosis grade and UDFF for prevalent carotid plaque. All models included age, sex, statin therapy, and LDL-C as clinical covariates. Conventional ultrasonographic steatosis was entered as a four-category variable with absent steatosis as the reference. C-statistics were 0.633 for the base model, 0.657 after adding conventional ultrasonographic steatosis grade, 0.747 after adding UDFF, and 0.787 after adding both. The comparison is cross-sectional and does not establish prospective predictive utility.

**Supplementary Figure S5. Diagnostic plots for the primary linear regression model of CIMT.**

(A) Normal Q-Q plot of standardized residuals assessing residual normality. (B) Scatter plot of standardized residuals versus fitted values assessing homoscedasticity and model specification. The primary Model 2 included continuous UDFF, age, sex, statin therapy, and LDL-C.
